# RNF6 activates TGF-β1/c-Myb pathway to promote EMT in esophageal squamous cell carcinoma

**DOI:** 10.3389/fonc.2023.1081333

**Published:** 2023-02-09

**Authors:** Jingge Cheng, Kun Wu, Qian Yang, Ziming Zhu, Hongye Zhao

**Affiliations:** ^1^ The Department of Thoracic Surgery, The Fourth Hospital of Hebei Medical University, Shijiazhuang, China; ^2^ The Department of Anesthesiology, The Fourth Hospital of Hebei Medical University, Shijiazhuang, China; ^3^ The Department of Thoracic Surgery, Han Dan Central Hospital, Handan, China; ^4^ The Department of Thoracic Surgery, The First Hospital of Xingtai, Xingtai, China; ^5^ The Department of Dermatology, The Fourth Hospital of Hebei Medical University, Shijiazhuang, China

**Keywords:** esophageal squamous cell carcinoma, RNF6, TGF-β1 signaling pathway, proliferation, invasion

## Abstract

**Objective:**

This study aimed to investigate RING-Finger Protein 6 (RNF6) expression in esophageal squamous cell carcinoma (ESCC) cells and whether it affects cell proliferation, invasion, and migration by regulating the TGF-β1/c-Myb pathway.

**Methods:**

TCGA database was used to analyze RNF6 expression in normal tissues and esophageal cancer tissues. Kaplan–Meier method was used to examine the correlation between RNF6 expression and patient prognosis. SiRNA interference vector and RNF6 overexpression plasmid were constructed, and RNF6 was transfected into Eca-109 and KYSE-150 esophageal cancer cell line. *In vitro* scratch assay and Transwell assay were conducted to investigate the effects of RNF6 on the migration and invasion of Eca-109 and KYSE-150 cells. RT-PCR detected the expression of Snail, E-cadherin, and N-cadherin, and TUNEL detected the apoptosis of cells.

**Results:**

RNF6 up-regulation promoted the progression of esophageal cancer and predicted poor prognosis. RNF6 also enhanced the migration and invasion of ESCC cells *in vitro*. RNF6 silencing inhibited the migration and invasion of ESCC cells. TGF-β inhibitors reversed the oncogenic effects of RNF6. RNF6 regulated the migration and invasion of ESCC cells by activating the TGF-β pathway. RNF6/TGF-β1 promoted esophageal cancer progression through c-Myb.

**Conclusion:**

RNF6 promotes the proliferation, invasion, and migration of ESCC cells possibly by activating the TGF-β1/c-Myb pathway and affects the progression of ESCC.

## Introduction

Esophageal carcinoma is one of the common malignant tumors of the digestive tract (1) and has low rates of early diagnosis, prognosis, and mortality (2). Adenocarcinoma and squamous cell carcinoma are the main histological types of esophageal cancer, and more than 90% of esophageal cancer cases in China are classified as esophageal squamous cell carcinoma (ESCC) (3). ESCC is characterized by high morbidity and low survival rate. Studying the key molecules and signal transduction mechanism of ESCC can provide new ideas for its early diagnosis and treatment (4, 5).

Epithelial–mesenchymal transition (EMT) is the morphological and genetic change of epithelial cells from tightly connected epithelial phenotype cells to mesenchymal phenotype cells with migration and invasion abilities (5, 6). EMT is involved in embryonic development, normal physiology, and many pathological processes, making it an important event in tumor invasion and metastasis (7). EMT plays an important role in the invasion and metastasis of ESCC and is closely related to the prognosis of patients (4, 8, 9). Transforming growth factor β (TGF-β) is an important inducer of tumor cell EMT (10). Under the mediation of TGF-β, epithelial tumor cells acquire mesenchymal phenotypes and lose epithelial phenotypes, leading to improvement in their invasion and migration abilities (11) and thus accelerating the spread and development of tumors (12). c-Myb oncogene regulates cell growth, proliferation, and differentiation (13). The amplification of c-Myb gene or the overexpression of c-Myb protein can promote tumor cell growth. TGF-β1 regulates the expression of the transcriptional activator c-Myb in most cells (12).

RNF6 is an E3 ligase in the ubiquitin–proteasome system (14). Its gene sequence is 2058 bp, and it encodes 685 amino acids (15). RNF6 has a C-terminal RING-H2 finger structure and an N-terminal helical domain. This gene stabilizes the adrenal receptor (AR) by inducing the non-hydrolytic ubiquitination of AR through its C-terminal RING-H2 finger structure (16). ARA54 is recruited to upregulate AR-mediated gene transcription (17). RNF6 also promotes prostate cancer cell growth (16); however, its mechanism in ESCC is unclear.

This study aimed to detect RNF6 expression in esophageal cancer. To investigate the effect of RNF6 on the proliferation, invasion and migration of esophageal cancer cells and whether this mechanism is realized through the regulation of TGF-β/c-Myb pathway, so as to provide a new experimental basis for clinical treatment of esophageal cancer.

## Methods

### Bioinformatics analysis

UALCAN online (http://ualcan.path.uab.edu/analysis.html) is biological analysis tool. Data were obtained from a wide range of sources, including RNA sequencing expression data from tumor and normal samples from the TCGA database. All data were standardized for subsequent analysis. UALCAN database was used to verify the differential expression of core genes between esophageal cancer and normal tissues, and P<0.05 was considered to be statistically significant. LinkedOmics database (http://www.linkedomics.org) was employed to analyze the correlation of RNF6 genes and the functional enrichment analysis of co-expressed genes.

### Cell culture

Human esophageal cancer cell lines Eca-109 and KYSE-150 (provided by Shanghai Cell Bank, Chinese Academy of Sciences) were cultured in RPMI-1640 medium containing 10% fetal bovine serum. Conventional subculture was conducted in a 5% CO_2_ incubator at 37°C with saturated humidity.

### Cell transfection

The cells were inoculated in six-well plates and cultured at 37°C and 5% CO_2_ for 12 h. When the cell fusion reached 80%, a transfection experiment was carried out following the operation instructions of the transfection reagent. The transfection reagents containing pcDNA.3.1-RNF6 (3 μg), pcDNA.3.1 (3 μg), and RNF6 siRNA or unrelated control siRNA (3 μg) were mixed with 200 μL of RPMI-1640 medium without serum and added into each well. The transfection efficiency was detected by QRT-PCR after incubation at 37°C with 5% CO_2_ for 48 h. Human RNF6 (si-RNF6) sequences were as follows: sense 5′-CCCGAACAAUGGAGAGUUUTT-3′ and antisense 5′-AAACUCUCCAUUGUUCGGGTT-3′. Negative scramble control sequences (si-NC) were as follows: sense 5′-UUCUCCGAACGUGUCACGUTT-3′ and antisense 5′-ACGUGACACGUUCGGAGAATT-3′.

### qRT-PCR

Cell lysate was added to each group of cells, and total cell RNA was extracted. PCR amplification was performed after reverse transcription into cDNA. Reaction conditions were as follows: 95°C for 3 min; 95°C for 5 s, 56°C for 10 s, and 72°C for 25 s for 40 cycles; and 65°C for 5 s. The relative quantification method (18) was applied to determine differences in the mRNA expression of RnF6 in each group using the following primers: RNF6-Forward: 5′-AGAAGATGGCAGCAAGAGCG-3′, RNF6-Reverse: 5′-TCAAGTCAGGCTGAGATGCTAGT-3′; GAPDH-Forward: 5′-GAAGGTGAAGGTCGGAGTC-3′; and GAPDH-Reverse: 5′-GAAGATGGTGATGGGATTTC-3′. GAPDH was selected as the internal reference gene, and 2^-ΔΔCt^ was used to represent the mRNA expression fold relationship of RNF6 in each group.

### Scratch test

Cells were seeded into six-well plates at a cell density of 5×10 (5) per well. FBS-free medium was added, and a straight line parallel to the middle part of each hole was drawn with 1 mL sterile elongated spear head in the same direction. Scratches were observed under a mirror, photographed, and marked. The scratches of each group were observed under a microscope at 0 and 24 h.

### Transwell experiment

Each Transwell membrane was spread with 30 μL of matrix glue and left overnight. On the next day, the cell suspension was added and the membrane was inoculated. A medium without FBS was added to the upper layer, and a medium containing 10% FBS was added to the lower layer. After incubation for 24 h, the cells of the upper compartment were removed. 0.1% crystal violet was dyed at room temperature for 20min. The cells were counted under a microscope.

### TUNEL experiment

In brief, 2×10 (5) Eca-109 and KYSE-150 cells were inoculated on six-well plates (two cover slides were placed in each well). The culture medium was discarded, and the plates were washed with PBS for three times and fixed with 4% paraformaldehyde. The cover glass was removed after successive treatment with 3% H_2_O_2_-methanol and 0.2% Triton X-100. TUNEL staining was performed following the *In Situ* Cell Death Detection Kit instructions. The number of green fluorescent cells was observed under an inverted fluorescence microscope (Leica, Germany), and the apoptotic cell rate was calculated.

### Western blot assay

Cells were collected and washed with PBS once. Total proteins were extracted from cell samples using NP-40 lysis buffer containing 40μg/mL protease inhibitor and phosphatase inhibitor. The protein concentration was determined using the BCA protein determination kit (Beyotime Biotechnology Co., LTD. Shanghai, China) according to the manufacturer’s instructions. The same amount of 40 μg protein was electrophoretic in SDS-PAGE 10% gel. It was then transferred to the PVDF membrane (Millipore, USA). After being closed with 5% skim milk for 1h, they were incubated with primary antibody E-cadherin (Abcam, 1:1000) and N-cadherin (Abcam, 1:1000) at 4°C overnight. TBST film washing 3 times for 10min each time. The secondary antibody conjugated with horseradish peroxidase was incubated at room temperature for 1h. TBST washed the film 3 times, 10min each time. Protein bands were observed by ECL assay kit.

### Statistical analysis

SPSS 22.0 software was employed for statistical analysis, and values were expressed as (mean ± SEM). T test was used for comparison between two groups, and one-way ANOVA was used for comparison between multiple groups. Kaplan–Meier method was applied for survival analysis. P<0.05 was considered statistically significant.

## Results

### RNF6 expression was increased in ESCC cells

**Figure 1:**
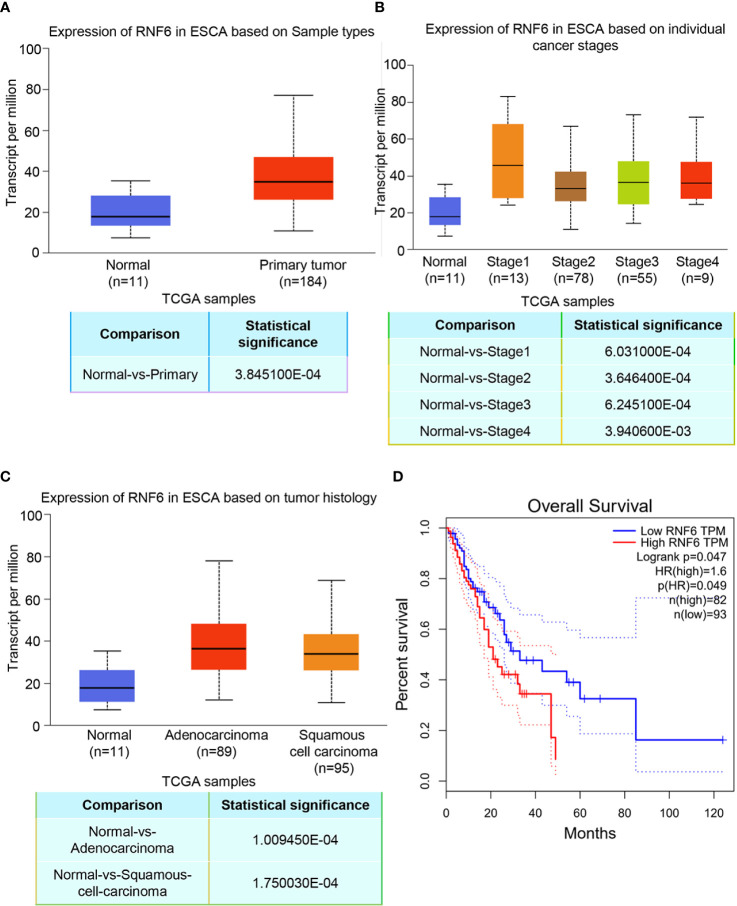
Upregulation of RNF6 expression promotes esophageal cancer progression and predicts poor prognosis. **(A)** TCGA analysis of the relative levels of RNF6 in adjacent normal tissues and esophageal cancer tissues. **(B)** Relative expression of RNF6 in esophageal cancer stage I, II, III/IV tissues. **(C)** RNF6 levels in ESCA of different pathological types. **(D)** The OS of 175 cases of esophageal cancer was analyzed by Kaplan-Meier, and the log-rank test was performed according to the RNF6 level.

TCGA data analysis showed that RNF6 expression significantly increased in human esophageal cancer group compared with the paracancer control group (**Figure 1A**, P<0.01). **Figure 1B** depicts RNF6 expression in different tumor stages. Compared with that in normal cells, RNF6 was highly expressed in Stage1 cells. **Figure 1C** shows RNF6 expression in ESCA based on tumor histology. RNF6 was highly expressed in adenocarcinoma and squamous cell carcinoma. Survival analysis revealed that patients with high RNF6 expression (n=82) had a shorter survival than patients with low RNF6 expression (n=93) (**Figure 1D**, P<0.05).

### RNF6 affected the invasion and migration of ESCC cells Eca-109 and KYSE-150

**Figure 2:**
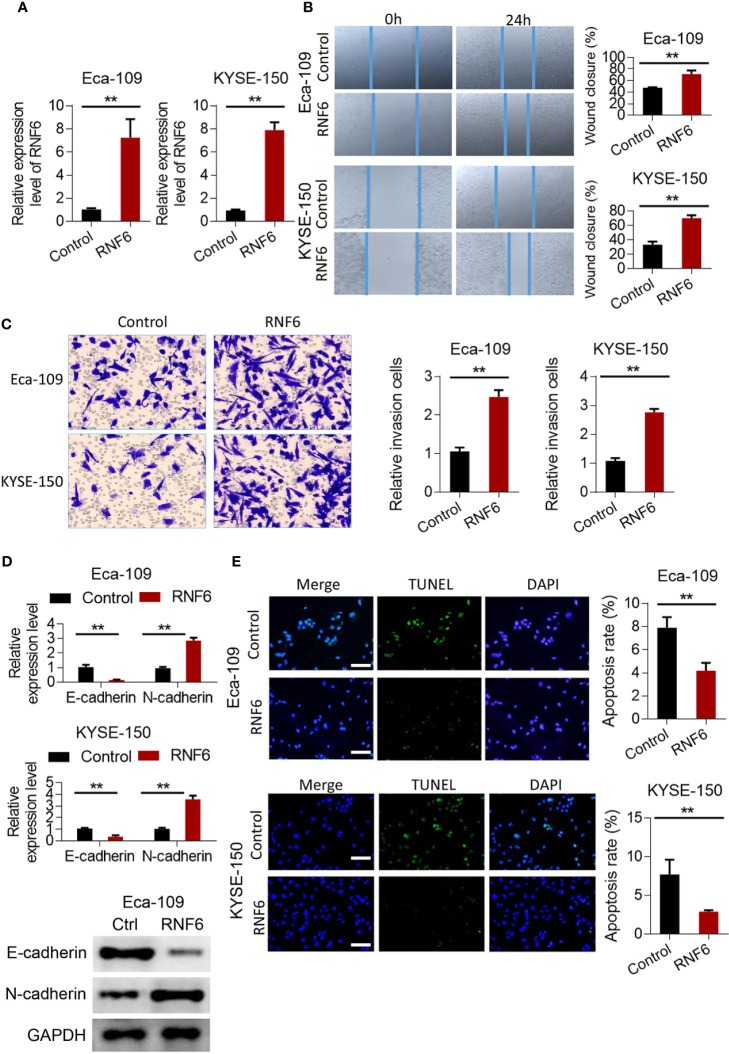
RNF6 promotes migration and invasion of esophageal cancer cells *in vitro*. **(A)** Overexpression efficiency assay. **(B)** Effect of overexpression of RNF6 on wound healing. **(C)** Effect of overexpression of RNF6 on cell invasion. **(D)** Changes in E-cadherin and N-cadherin levels after RNF6 upregulation. **(E)** Cell apoptosis was detected by TUNEL after overexpression of RNF6. Scale bar: 50 µm. n=3, **P < 0.01.

Scratch test results showed that after the stable overexpression of RNF6 (**Figure 2A**), the Eca-109 and KYSE-150 cells showed significantly enlarged cell scratch healing area and enhanced migration ability (**Figure 2B**, P<0.01). Transwell invasion assay revealed that the number of tumor cells below the Transwell compartment membrane significantly increased in the overexpressed RNF6 group, and the invasion ability of cells was significantly enhanced (P<0.01, **Figure 2C**). **Figure 2D** illustrates the changes of E-cadherin and N-cadherin levels after RNF6 upregulation (Eca-109 and KYSE-150 cells). RNF6 overexpression inhibit E-cadherin but promoted N-cadherin. Apoptosis was detected by TUNEL after RNF6 overexpression. Compared with that of the control group, the apoptosis degree of RNF6 overexpressed group was significantly decreased (P<0.01, **Figure 2E**).

### Silencing RNF6 inhibited the migration and invasion of esophageal cancer cells

**Figure 3:**
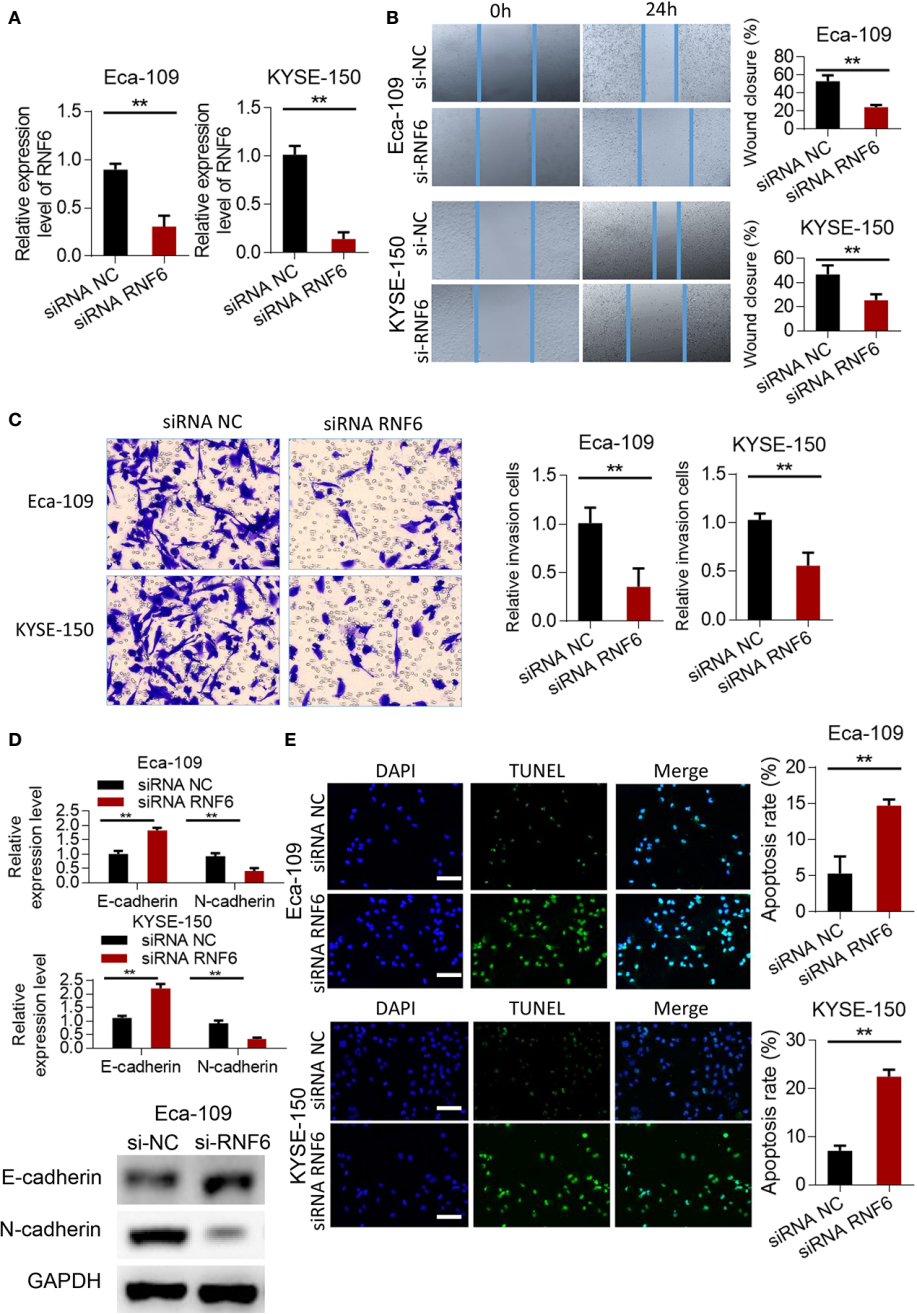
Silencing of RNF6 inhibits the migration and invasion of esophageal cancer cells. **(A)** Silencing efficiency assay. **(B)** Effect of overexpression of RNF6 on wound healing. **(C)** Effect of overexpression of RNF6 on cell invasion. **(D)** Changes in E-cadherin and N-cadherin levels after RNF6 upregulation. **(E)** Cell apoptosis was detected by TUNEL after overexpression of RNF6. Scale bar: 50 µm. n=3, **P < 0.01, Student’s T test analysis.

RNF6 expression in the siRNF6 group was significantly lower than that in the non-spe group (**Figure 3A**, P<0.01). *In vitro* scratch test showed that the migration ability of siRNF6 group was significantly weakened and the cell healing area was small (**Figure 3B**). Transwell assay showed that the number of invasive cells was reduced in the siRNF6 group (**Figure 3C**). qRT-PCR analysis of E-cadherin and N-cadherin showed that E-cadherin was up-regulated in the in siRNF6 group compared with that in the control group. Meanwhile, the expression of N-cadherin decreased (**Figure 3D**). TUNEL results showed that the apoptosis rate of siRNF6 group was significantly increased compared with that of the control group (P<0.01, **Figure 3E**).

### Co-expressed genes and functional enrichment analysis of RNF6 in esophageal cancer

**Figure 4:**
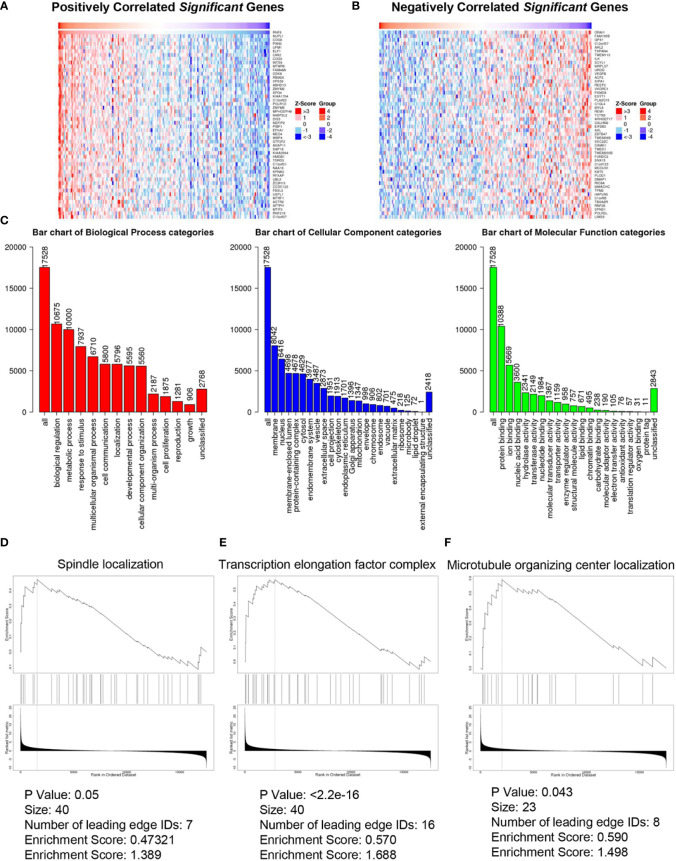
Co-expressed genes and functional enrichment analysis of RNF6 in esophageal cancer. **(A)** Heatmap analysis of genes that are positively correlated with RNF6 co-expression. **(B)** Heatmap analysis of negatively correlated genes with RNF6 co-expression. **(C)** Functional enrichment analysis of RNF6 co-expression. **(D–F)**. RNF6 co-expressed genes affect Spindle localization, Transcription elongation factor complex and Microtubule organizing center localization in esophageal cancer cells.

**Figures 4A, B** show the heatmap analysis of RNF6 co-expressed genes, respectively. The functions of RNF6 co-expressed genes were correlated with tumor proliferation, invasion and metastasis, and EMT. Functional enrichment analysis of RNF6 co-expressed genes revealed that RNF6 mainly affected cell communication, cellular component remodeling, and cell proliferation. It also affected the cytoskeleton, extracellular matrix, and microfilament microtubule composition (**Figure 4C**). **Figures 4D–F** show that RNF6 co-expressed genes affected spindle localization, transcription elongation factor complex, and microtubule organizing center localization of esophageal cancer cells. One of the hallmarks of EMT is a change in morphology, from pebble-like to fusiform cells. Cell polarity loss, skeletal changes, infiltration and migration ability increased. In addition, the EMT process can be triggered by a variety of signals, such as transcription factors and other oncogenic genes. Therefore, RNF affects the EMT of esophageal cancer.

### TGF-β inhibitors reversed the carcinogenic effect of RNF6

**Figure 5:**
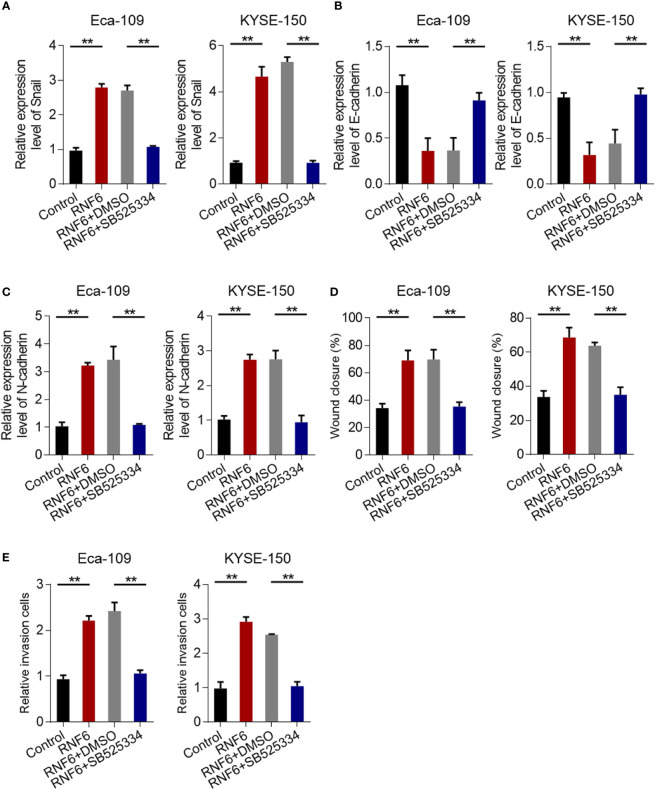
TGF-β inhibitors reverse the cancer-promoting effects of RNF6. **(A)** qPCR analysis of Snail mRNA levels in Eca-109 and KYSE-150 cells overexpressed with RNF6 or treated with SB525334 (a TGF-β inhibitor). **(B)** qPCR analysis of E-cadherin mRNA levels in Eca-109 and KYSE-150 cells overexpressed with RNF6 or treated with SB525334. **(C)** qPCR analysis of N-cadherin mRNA levels in Eca-109 and KYSE-150 cells overexpressed with RNF6 or treated with SB525334. **(D)** Effects of SB525334 and RNF6 upregulation on cell migration. **(E)** Effects of SB525334 and RNF6 upregulation on cell invasion. n=3, **P < 0.01, ANOVA analysis.

The results confirmed that RNF6 can promote the invasion and migration of esophageal cancer cells. Further study was conducted to determine the regulatory mechanism and whether TGF-β signaling pathway can achieve the above functions. qRT-PCR results showed that Eca-109 and KYSE-150 cells with RNF6 overexpression exhibited significantly up-regulated Snail mRNA expression. After treatment with TGF-β specific inhibitor SB525334, the Snail expression was down-regulated (**Figure 5A**). After RNF6 overexpression in Eca-109 and KYSE-150 cells, E-cadherin was significantly decreased and N-cadherin was up-regulated. After SB525334 treatment, E-cadherin was up-regulated and N-cadherin expression was down-regulated (**Figures 5B, C**). Compared with those in the RNF6 + DMSO group, the migration and invasion abilities of Eca-109 and KYSE-150 cells in the RNF6 + SB525334 group were significantly reduced (**Figures 5D, E**, P <0.01).

### RNF6 regulated the migration and invasion of ESCC cells by activating the TGF-β pathway

**Figure 6:**
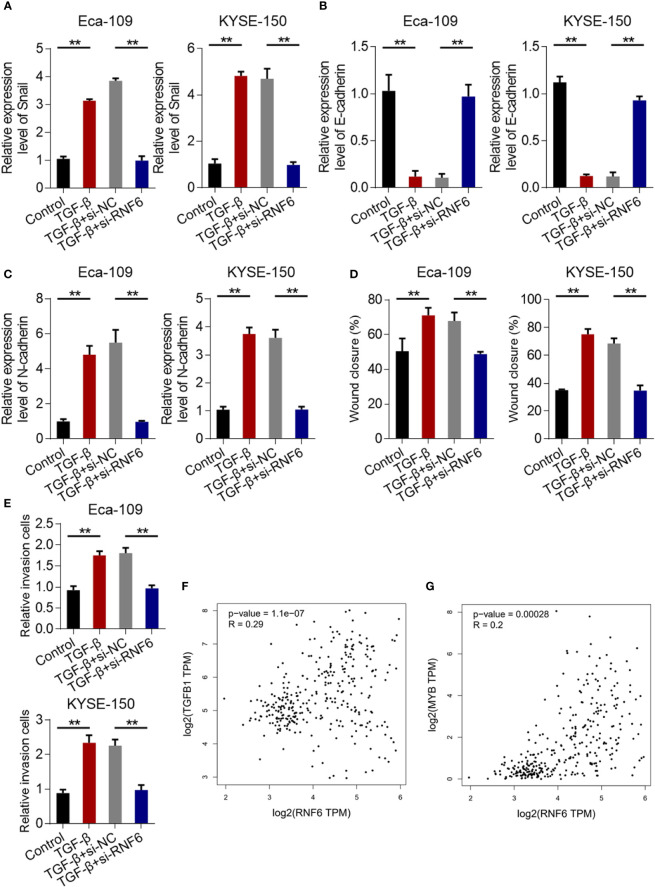
RNF6 regulates the migration and invasion of esophageal cancer cells by activating the TGF-β pathway. **(A)** qPCR analysis of Snail mRNA levels in Eca-109 and KYSE-150 cells treated with silenced RNF6 or TGF-β1. **(B)** qPCR analysis of E-cadherin mRNA levels in Eca-109 and KYSE-150 cells treated with silenced RNF6 or TGF-β1. **(C)** qPCR analysis of N-cadherin mRNA levels in Eca-109 and KYSE-150 cells treated with silenced RNF6 or TGF-β1. **(D)** Effects of silencing RNF6 or TGF-β1-treated Eca-109 and KYSE-150 cells on migration. **(E)** Effects of silencing RNF6 or TGF-β1 treatment on invasion of Eca-109 and KYSE-150 cells. n=3, **P < 0.01, ANOVA analysis. **(F)** The co-expression correlation between RNF6 and TGF-β. **(G)** The co-expression correlation between RNF6 and c-Myb.

Compared with that in the control group, the Snail mRNA of Eca-109 and KYSE-150 cells in the TGF-β group was up-regulated. Compared with that in the TGF-β + si-NC group, the Snail mRNA in the TGF-β + si-RNF6 group was significantly reduced (**Figure 6A**). After TGF-β treatment, E-cadherin was down-regulated and N-cadherin was up-regulated, suggesting that TGF-β promoted EMT in ESCC. Compared with TGF-β, E-cadherin was up-regulated and N-cadherin was down-regulated in the TGF-β + si-RNF6 group (**Figures 6B, C**). Compared with those in the TGF-β + si-NC group, the migration and invasion abilities of Eca-109 and KYSE-150 cells in the TGF-β + si-RNF6 group were significantly reduced (**Figures 6D, E**, P <0.01). **Figures 6F, G** show the co-expression correlation between RNF6 and TGF-β and c-Myb. RNF6 exhibited a positive correlation with TGF-β and c-Myb and its co-expression in esophageal cancer.

### RNF6/TGF-β1 promoted the progression of esophageal cancer through c-Myb

**Figure 7:**
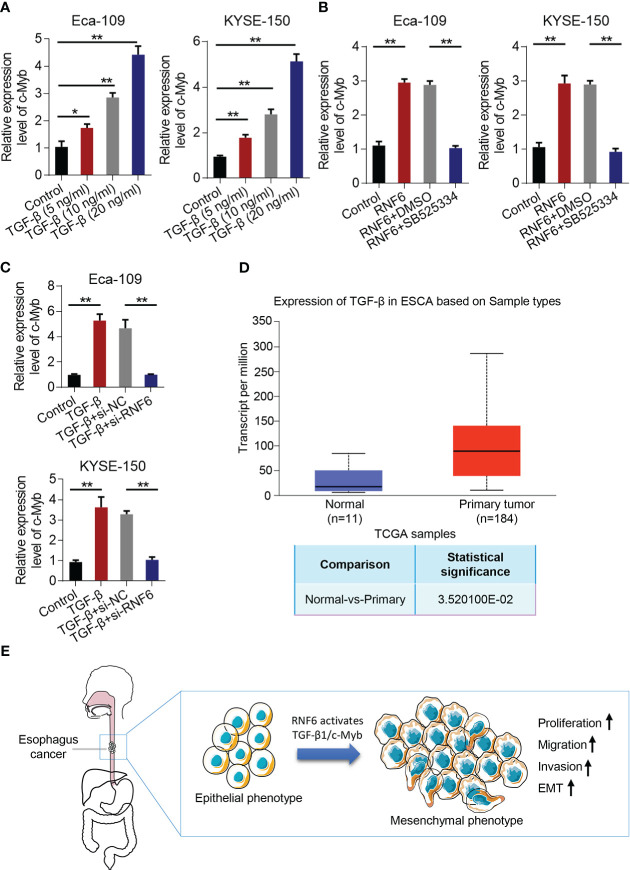
RNF6/TGF-β1 promotes esophageal cancer progression through c-Myb. **(A)** The level of c-Myb in cells increased after the concentration of TGF-β1 was increased. **(B)** qPCR analysis of c-Myb mRNA levels in RNF6 overexpressed or SB525334 (TGF-β inhibitor)-treated Eca-109 and KYSE-150 cells. **(C)** qPCR analysis of c-Myb mRNA levels in Eca-109 and KYSE-150 cells treated with silenced RNF6 or TGF-β1. **(D)** TCGA analysis of the relative levels of TGF-β in adjacent normal tissues and esophageal cancer tissues. **(E)** Mechanism diagram of RNF6 promoting EMT. n=3, *P <0.05; **P < 0.01, ANOVA analysis.

Compared with that in the control group, c-Myb mRNA increased in the TGF-β group (**Figure 7A**). qPCR was conducted to analyze c-Myb mRNA levels in Eca-109 and KYSE-150 cells treated with RNF6 overexpression or SB525334 (TGF-β inhibitor). c-Myb mRNA was significantly up-regulated in Eca-109 and KYSE-150 cells after the stable overexpression of RNF6. After treatment with TGF-β specific inhibitor SB525334, c-Myb mRNA was down-regulated (**Figure 7B**). Compared with that in the control group, the c-Myb mRNA of Eca-109 and KYSE-150 cells in the TGF-β group was up-regulated. Compared with that in the TGF-β + si-NC group, the c-Myb mRNA in the TGF-β + si-RNF6 group was significantly decreased (**Figure 7C**). TCGA was used to analyze the relative levels of TGF-β in paracancer normal tissues and esophageal cancer tissues. TCGA data analysis results showed that TGF-β expression was significantly increased in human esophageal cancer compared with that in the paracancer control group (**Figure 7D**). **Figure 7E** reveals the molecular mechanism of RNF6 in promoting EMT in esophageal cancer.

## Discussion

ESCC is one of the most common gastrointestinal malignancies in the world (19). Although radical ESCC resection combined with chemotherapy has been widely used for treatment, the 5-year survival rate is low because most patients are already in advanced stage upon diagnosis (20). Reducing cell proliferation and inducing cell apoptosis will greatly improve the therapeutic effect against ESCC (1). With the continuous improvement of biotechnology, molecular drug targeted therapy is gradually being used in clinical diagnosis and treatment because of its accurate localization and improved survival rate (21). Therefore, finding new ESCC molecular targets is the key to the early diagnosis and treatment of ESCC (6).

RNF6 regulates the transcriptional activity and specificity of AR, thereby promoting the growth of prostate cancer cells (16). Therefore, RNF6 is closely associated with the growth of prostate cancer cells (22). In addition, RNF6 neurons in the hippocampus are closely related to the growth of nerve cells’ axons (23). RNF6 is high in cisplatin-resistant lung cancer cell lines, indicating that it may also be closely related to drug resistance (23, 24). However, the function of RNF6 in ESCC has not been thoroughly studied. To verify the biological function of RNF6 in ESCC cells, this study explored RNF6 expression in esophageal cancer at the clinical level. Cell lines Eca-109 and KYSE-150 were treated with RNF6 interference. Results showed that after RNF6 inhibition, the scratch area of Eca-109 and KYSE-150 cells was small, their migration ability decreased (P<0.05), and the number of tumor cells below the Transwell compartment membrane significantly decreased (P<0.05). In addition, the proliferation and invasion of RNF6 cells were significantly inhibited by silencing RNF6. These results suggested that RNF6 could affect the proliferation, invasion, and migration of ESCC cells.

TGF-β is a functional protein that can control the proliferation, differentiation, and apoptosis of various cell types (25). It plays a complex and important role in the occurrence and development of tumors. As tumors develop, TGF-β is overexpressed in the tumor microenvironment due to the accumulation of related gene mutations and epigenetic modifications (26). TGF-β also induces the EMT of tumor epithelial cells through Smad and non-Smad pathways and promotes tumor cell migration and metastasis (27). It can promote the regeneration of tumor blood vessels and inhibit the body’s immune system for tumor killing effect (28). Through TCGA data analysis, we found that in ESCA, RNF6 had a positive correlation with TGF-β co-expression. TGF-β/c-Myb axis promotes epithelial ovarian cancer progression reported by Lin et al (29). Our further analysis found that RNF6 and c-Myb also had positive co-expression correlation in ESCA. Therefore, we chose to explore the effects of RNF6 on TGF-β1/c-Myb signaling pathway. This study showed that RNF6 suppression significantly reduced c-Myb protein expression. Moreover, treatment with TGF-β inhibitor SB525334 significantly reversed the carcinogenic effect of RNF6. These results suggested that RNF6 can regulate the expression of c-Myb by regulating TGF-β. Down-regulating c-Myb can inhibit the proliferation and promote the apoptosis of ESCC cells (30). Therefore, inhibiting RNF6 can suppress the proliferation and promote apoptosis of ESCC cells by down-regulating c-Myb.

## Conclusion

RNF6 expression was significantly increased in ESCC. Down-regulating RNF6 significantly inhibited the proliferation and promoted apoptosis of ESCC cells. Silencing RNF6 can suppress TGF-β/c-Myb. RNF6 regulates c-Myb through TGF-β, thus regulating the proliferation and apoptosis of ESCC cells.

## Data availability statement

The datasets presented in this study can be found in online repositories. The names of the repository/repositories and accession number(s) can be found in the article/supplementary material.

## Ethics statement

Written informed consent was obtained from the individual(s) for the publication of any potentially identifiable images or data included in this article.

## Author contributions

HZ designed the study. JC, KW, and QY performed the experiments and analyzed the data. JC and ZZ performed the data analysis. JC wrote the initial draft of the paper, with contributions from all authors. All authors contributed to the article and approved the submitted version.
